# Enzymatic response of ryegrass cellulose and hemicellulose valorization introduced by sequential alkaline extractions

**DOI:** 10.1186/s13068-021-01921-1

**Published:** 2021-03-19

**Authors:** Shao-Fei Sun, Jing Yang, Da-Wei Wang, Hai-Yan Yang, Shao-Ni Sun, Zheng-Jun Shi

**Affiliations:** 1 Key Laboratory for Forest Resources Conservation and Utilization in the Southwest Mountains of China, Ministry of Education, Southwest Forestry University, Kunming, 650224 China; 2 Key Laboratory of State Forestry and Grassland Administration on Highly-Efficient Utilization of Forestry Biomass Resources in Southwest China, Southwest Forestry University, Kunming, 650224 China; 3 Beijing Key Laboratory of Lignocellulosic Chemistry, Beijing Forestry University, Beijing, 100083 China

**Keywords:** Ryegrass, Cellulose, Hemicelluloses structure, Enzymatic hydrolysis, Alkaline extraction

## Abstract

**Background:**

In view of the natural resistance of hemicelluloses in lignocellulosic biomass on bioconversion of cellulose into fermentable sugars, alkali extraction is considered as an effective method for gradually fractionating hemicelluloses and increasing the bioconversion efficiency of cellulose. In the present study, sequential alkaline extractions were performed on the delignified ryegrass material to achieve high bioconversion efficiency of cellulose and comprehensively investigated the structural features of hemicellulosic fractions for further applications.

**Results:**

Sequential alkaline extractions removed hemicelluloses from cellulose-rich substrates and degraded part of amorphous cellulose, reducing yields of cellulose-rich substrates from 73.0 to 27.7% and increasing crystallinity indexes from 31.7 to 41.0%. Alkaline extraction enhanced bioconversion of cellulose by removal of hemicelluloses and swelling of cellulose, increasing of enzymatic hydrolysis from 72.3 to 95.3%. In addition, alkaline extraction gradually fractionated hemicelluloses into six fractions, containing arabinoxylans as the main polysaccharides and part of *β*-glucans. Simultaneously, increasing of alkaline concentration degraded hemicellulosic polysaccharides, which resulted in a decreasing their molecular weights from 67,510 to 50,720 g/mol.

**Conclusions:**

The present study demonstrated that the sequential alkaline extraction conditions had significant effects on the enzymatic hydrolysis efficiency of cellulose and the investigation of the physicochemical properties of hemicellulose. Overall, the investigation the enzymatic hydrolysis efficiency of cellulose-rich substrates and the structural features of hemicelluloses from ryegrass will provide useful information for the efficient utilization of cellulose and hemicelluloses in biorefineries.

## Background

The global energy and financial crisis promote the development of renewable energies [1]. Bioethanol derived from lignocellulosic biomass is considered as an alternative energy to fossil oil due to its environmentally attractive and technologically feasible. Among the lignocelluloses, short rotation grasses are targeted as potential energy crops due to their abundance, availability, and high productivity [2–4]. In addition, grasses can be utilized as whole plants due to high percentage of total carbohydrates and comparatively less lignin content. Ryegrass is the most common bunch type of grass that widely used across the world as a forage and cover crop. The high abundance of ryegrass allows it to be a promising lignocellulosic feedstock for bioethanol production [5]. However, bioethanol production from grass is impeded by the recalcitrant structure of cell wall. Thus, an efficient pretreatment process is required to disrupt the intact structure of biomass and release carbohydrate polymers for further fermentation [3, 4].

Pretreatment of lignocelluloses is the process which removes hemicelluloses and lignin, reduces cellulose crystallinity, and increases accessibility of material for enzymes [6, 7]. The hemicelluloses in the plant cell wall have an inhibitory effect on the enzymatic hydrolysis of cellulose, since this component covers the cellulose microfibers and further forms a physical barrier to prevent the entry of enzymes [8]. Lignin can not only form a shielding layer, but also can non-productively and irreversibly adsorb cellulase, resulting in a very low enzymatic hydrolysis rate of cellulose [9, 10]. Among the pretreatment technologies, alkaline pretreatment is one of the major chemical pretreatments due to practical advantages such as low reaction temperature and pressure, no need for complicated reactors [11]. Up to now, alkaline pretreatment has been widely applied on lignocellulosic biomass with low lignin contents, such as agricultural wastes, herbaceous crops, and hardwoods. During alkaline pretreatment, hydrogen and covalent bonds (such as ester and ether bonds) are broken, resulting in alteration of lignin structure and disruption of crosslinks between hemicelluloses and other components [12]. The cleavage of these linkages facilitates dissociation of entire cell wall of lignocelluloses and solubilization of hemicelluloses and lignin, improving accessibility of lignocellulose. In addition, more hemicelluloses are dissolved during the alkaline pretreatment as compared to lignin and cellulose [13]. The removal of hemicelluloses is often correlated well with the increase of enzymatic hydrolysis of lignocellulosic biomass. Although hemicelluloses can be hydrolyzed into its component sugars, the pentoses, such as xylose and arabinose, in hemicelluloses are difficult to ferment to ethanol because of the lack of the natural microorganisms that metabolize xylose or arabinose [14]. Thus, hemicelluloses can be recovered by alkaline treatment for further utilization.

Hemicelluloses, the second most abundant structural polymers in lignocellulosic biomass, contain different types of sugars according to plant resources. Generally, glucouronoxylan (xylan) is found to be the principal constituent of the hemicelluloses in hardwood and agriculture residues, while galactoglucomannan is the principal component of softwood hemicelluloses [15–17]. Different from cellulose, hemicelluloses are branched heteropolysaccharides of several different neutral and acidic monosaccharides [18]. Apart from origin of hemicelluloses, extraction methods also affect the properties of hemicelluloses. Among different hemicelluloses extraction methods, alkaline and hot water extractions are the most popular processes. After extraction, the obtained hemicelluloses can used directly as natural polymers in industries or used as feedstock for producing platform chemicals [18]. For instance, the hemicelluloses can be partially hydrolyzed to produce xylo-oligosaccharides (XOS), which is considered as a prebiotic and widely used in health products due to its ability to improve the calcium absorption, reduce the cholesterol, promote the growth of probiotics, and lower the risk of colon cancer [19, 20].

In this study, cellulose-rich substrates and hemicellulosic fractions of ryegrass were gradually recovered by sequential alkaline extractions from delignified material. The effects of sequential alkaline treatments on chemical compositions and structural characteristics of the samples were analyzed by sugar analysis, Fourier transform infrared (FT-IR), and nuclear magnetic resonance (NMR) spectroscopy, respectively. In addition, the effect of sequential alkaline treatments on enzymatic hydrolysis of cellulose-rich substrate was also evaluated.

## Results and discussion

### Yields and chemical compositions of cellulose-rich substrates

Hemicellulosic compounds mutually adhered with cellulose microfibrils by hydrogen bonds and van der Waals forces, holding the stiff cellulose fibrils in place. However, hemicelluloses have been considered as major obstacle of physically penetrating and attacking the cellulose by cellulase in bioconversion process [21]. Aqueous alkaline treatment has been considered as an efficient process for hemicelluloses extraction. Yields and chemical compositions of cellulose-rich substrates obtained from sequential alkaline extractions are shown in Table 1. Cellulose-rich substrate (R_pulp_) obtained by delignification contained 47.8% glucan as the major sugar. Hemicellulosic compounds, including xylan (19.7%), arabinan (7.7%), galactan (2.6%), mannan (0.1%), galacturonic acid (2.3%), and glucuronic acid (0.3%), totally accounted 32.7% of the substrate. The chemical compositions of hemicellulosic compounds in delignified ryegrass indicated that arabinoxylans was the main compound of hemicellulosic fractions. This result was consistent with the chemical compositions of hemicellulosic fractions obtained from sequential alkaline extractions. Besides, 3.2% Klason lignin and 0.7% acid-soluble lignin were remained in the cellulose-rich substrate. After alkaline extraction, part of the hemicellulosic compounds and lignin were removed. As the alkaline concentration increased from 0.15 to 2.5%, the yields of solid cellulose-rich substrates also decreased from 73.0 to 27.7%. The contents of hemicellulosic compounds and lignin decreased from 30.3 to 19.2%, and from 2.3 to 0.7%, respectively. The residual hemicelluloses in substrates were xylans, which were the main compounds, and their contents decreased from 18.2 to 14.3%. The solubilization of hemicellulosic fractions was accompanied with increase of cellulose contents from 51.1 to 62.2%. Increasing of cellulose content is usually preferred for ethanol production due to the direct proportional relationship of ethanol yield and cellulose content of substrate [22]. These results were similar with the composition analysis of cellulosic samples obtained from sequential NaOH extractions of oat straw holocellulose [23]. However, a less amount of glucan in cellulose-rich substrate was observed after alkaline extraction in this study. It might be ascribed to the lower extraction temperature performed in this study. Besides, the dilute acid pretreatment of sugarcane bagasse before alkaline extraction also largely removes hemicellulosic fraction and releases higher content of cellulose in solid fraction than which in ryegrass cellulosic substrates [24].

**Table 1:** Yields and chemical components of cellulose-rich substrates obtained from sequential alkaline extractions of delignified ryegrass

Samples	Yield^a,c^ (%)	Substrates composition^b,c^ (%)								
		Ara	Gal	Glu	Xyl	Man	GalA	GluA	KL	ASL
R_pulp_	100.0 ± 0.6	7.7 ± 0.2	2.6 ± 0.0	47.8 ± 1.5	19.7 ± 0.6	0.1 ± 0.0	2.3 ± 0.0	0.3 ± 0.0	3.2 ± 0.1	0.7 ± 0.0
R_0.15%_	73.0 ± 3.3	7.1 ± 0.2	1.8 ± 0.0	51.1 ± 1.7	18.2 ± 0.6	0.1 ± 0.0	2.7 ± 0.0	0.4 ± 0.0	1.8 ± 0.0	0.5 ± 0.0
R_0.3%_	68.9 ± 3.2	6.6 ± 0.2	1.2 ± 0.0	52.7 ± 1.6	17.7 ± 0.5	0.1 ± 0.0	2.4 ± 0.0	0.3 ± 0.0	1.8 ± 0.0	0.4 ± 0.0
R_0.5%_	63.9 ± 2.9	5.1 ± 0.1	1.2 ± 0.0	54.7 ± 1.8	17.0 ± 0.5	0.1 ± 0.0	2.1 ± 0.0	0.2 ± 0.0	1.7 ± 0.0	0.4 ± 0.0
R_1.0%_	58.4 ± 2.6	4.3 ± 0.1	1.0 ± 0.0	57.9 ± 1.8	16.7 ± 0.5	0.2 ± 0.0	0.5 ± 0.0	0.2 ± 0.0	1.6 ± 0.0	0.3 ± 0.0
R_1.5%_	50.8 ± 2.4	3.8 ± 0.1	1.0 ± 0.0	59.0 ± 1.8	16.1 ± 0.5	0.2 ± 0.0	0.4 ± 0.0	0.2 ± 0.0	0.9 ± 0.0	0.3 ± 0.0
R_2.5%_	27.7 ± 1.3	3.3 ± 0.1	0.8 ± 0.0	62.2 ± 2.0	14.3 ± 0.4	0.2 ± 0.0	0.4 ± 0.0	0.2 ± 0.0	0.4 ± 0.0	0.3 ± 0.0

^a^Yields of cellulose-rich substrates obtained from the delignified ryegrass by sequential alkaline extractions, calculated as [(the weight of cellulose-rich substrate obtained after each alkaline extraction)/(the weight of the delignified ryegrass used for sequential alkaline extractions)] × 100%;

^b^Ara, arabinan; Gal, galactan; Glu, glucan; Xyl, xylan; Man, mannan; GalA, galacturonic acid; GluA, glucuronic acid; ASL, acid-soluble lignin; KL, Klason lignin

^c^The values are mean ± SD of three parallel determinations

### FT-IR spectra analysis of cellulose-rich substrates

**Fig. 1:**
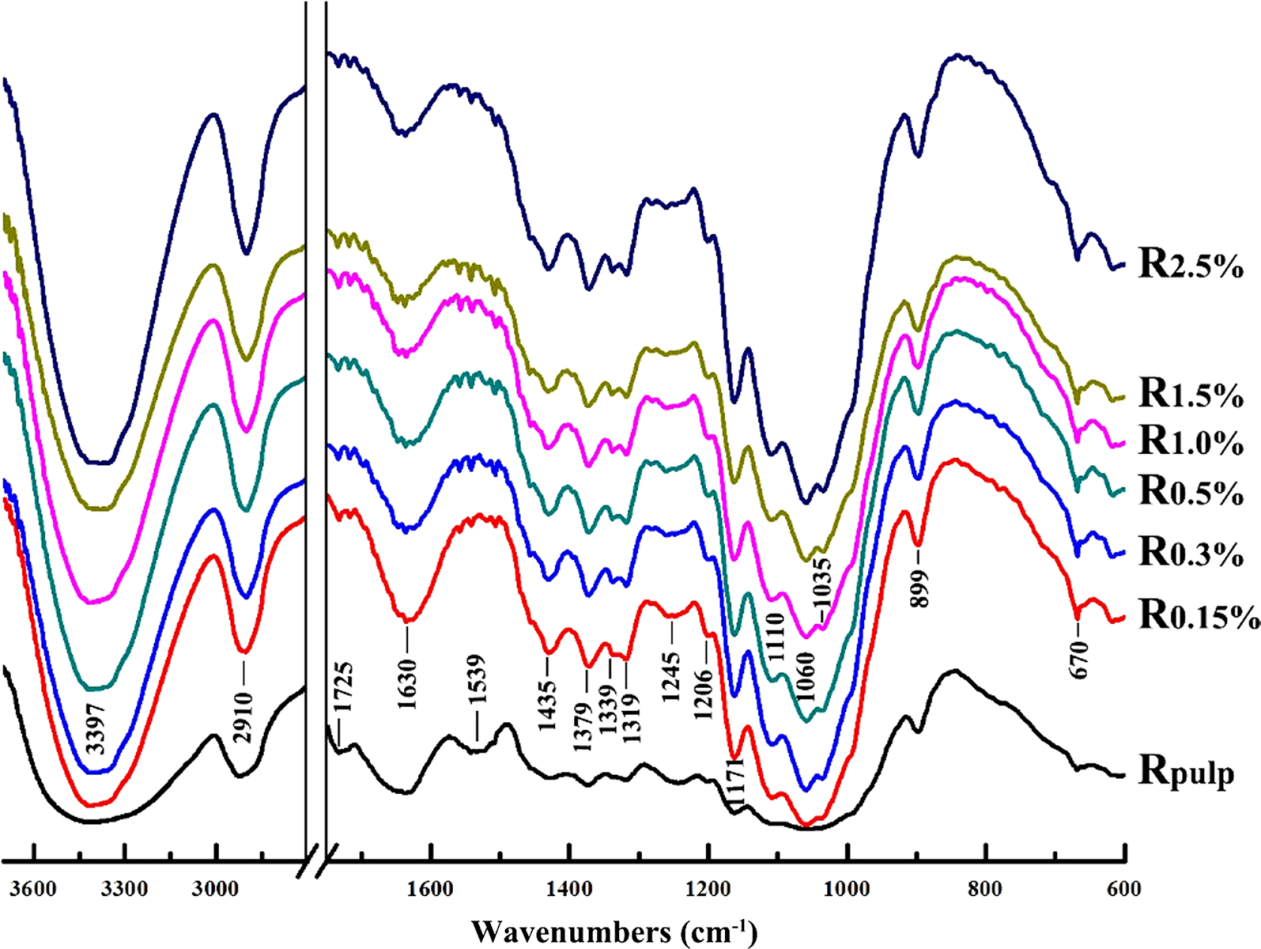
FT-IR spectra of the delignified ryegrass (R_pulp_) and the cellulose-rich substrates (R_0.15%_, R_0.3%_, R_0.5%_, R_1.0%_, R_1.5%_, and R_2.5%_) obtained by sequential alkaline treatments of the delignified ryegrass

Under alkaline condition, the ester linkages in lignocellulose can be cleaved at relatively high temperature [25]. IR spectroscopy is a widely used to determine functional groups of complex polymers. FT-IR spectra of cellulose-rich substrates are shown in Fig. 1. The stretching vibration of –OH groups in substrates is observed at 3397 cm^−1^. The bands at about 1319, 1245, and 1206 cm^−1^ are due to the in-plane bending of –OH. The bands at 2910 and 1379 cm^−1^ are assigned to C–H stretching and C–H bending along the chain, respectively. The intense absorption band at 1630 cm^−1^ corresponds to the bending mode of the absorbed water. The attributions of the main adsorptions are characteristic of glycosidic structures at 1171, 1110, 1060, and 1035 cm^−1^ for antisymmetric bridge C–O–C and C–O stretching, respectively.

**Fig. 2:**
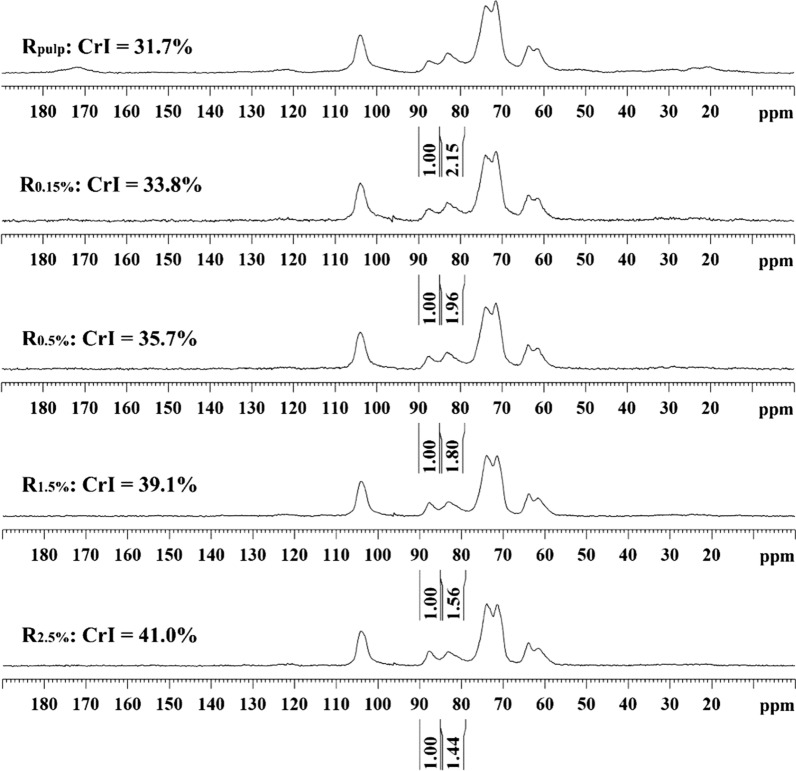
CP/MAS ^13^C-NMR spectra of the delignified ryegrass (R_pulp_) and the cellulose-rich substrates (R_0.15%_, R_0.5%_, R_1.5%_, and R_2.5%_) obtained by sequential alkaline treatments of the delignified ryegrass

A small band at about 899 cm^−1^ in the spectra is characteristic of C_1_ group of frequency/antisymmetric out-of-plane ring stretching due to *β*-glycosidic linkages. Although the spectral pattern of the samples was similar, the band (1725 cm^−1^) assigned for C=O stretching of acetyl groups in the spectrum of delignified ryegrass (R_pulp_) disappeared in the spectra of samples after alkaline extraction. This result indicated the deacetylation of the substrates under alkaline conditions. Pretreatment of corn stalk with 0.5% KOH solution at 30 °C for 24 h also obtains 91.34% deacetylation [26]. The disappearance of ester bonds in FT-IR spectra is consisted with the results observed in solid NMR spectra of cellulose-rich substrates (Fig. 2). In addition, the signal at around 1539 cm^−1^ in spectrum of R_pulp_ is assigned to the residual lignin (3.9%) in ryegrass holocellulose.

### Crystallinity analysis of cellulose-rich substrates

Solid-state NMR methodologies are particularly useful for studying structural characteristics of lignocellulose and individual plant cell wall components due to the fact that they can provide much chemical information and ultrastructural details [27]. ^13^C CP/MAS is one of the modern solid-state NMR methodologies; it can be used for a qualitative identification of the main chemical and structural changes taking place in the samples as a consequence of the pretreatments. CP/MAS spectra of cellulose-rich substrates obtained from sequential alkaline extractions are shown in Fig. 2. The signals between 60 and 110 ppm are singled to carbohydrates. The signal at about 105 ppm origins from C_1_ groups of cellulose. The overlapping signals in the region of 70–80 ppm are assigned to C_2_, C_3_, and C_5_ of cellulose. In the spectra of cellulose, the amorphous carbons of C_4_ are represented by a fairly broad signal from 80 to 85 ppm, while crystalline carbons of C_4_ generate a sharper resonance from 85 to 92 ppm. Two phases of C_6_ cellulose are found at about 63 and 69 ppm, respectively. The peaks around 21 and 172 ppm in the spectrum of R_pulp_ origin for methyl and carboxylic carbons of acetyl groups attached to the hemicellulosic fractions. After alkaline extraction, the disappearance of these peaks in the spectra of cellulose-rich substrates indicated the cleavage of bonds between acetyl groups and backbone during alkaline extraction.

Crystallinity index (CrI) is an important characteristic affecting the enzymatic hydrolysis of cellulose. The C_4_ peak in the carbon spectrum of cellulose is the most commonly utilized peak used to extract ultrastructural information, such as crystalline domains [28]. During the alkaline treatment, alkali molecule can penetrate into the cellulose macromolecule and disrupt the hydrogen bonds between intra- and inter-molecule chains, thereby changing the ultrastructure of cellulose. The effects of sequential alkaline extractions on ordered structure of cellulose are shown as crystallinity index in CP/MAS spectra, which are calculated as the peak area ratio of crystalline to total of C_4_ signals. After alkaline extraction, the peak intensity for amorphous cellulose decrease, introducing an increase of cellulose-rich substrates crystallinity index (31.7, 33.8, 35.7, 39.1, and 41.0%). The increment of crystallinity index of cellulose was ascribed to the fact that alkaline treatments resulted in greater hydrolyzation of amorphous regions than crystalline regions and peeling reaction of the amorphous regions in cellulose [11]. In addition, an increase of crystalline index of the cellulose residue was also due to the removal of amorphous hemicelluloses from the pulp.

### Enzymatic hydrolysis of cellulose-rich substrates

**Fig. 3:**
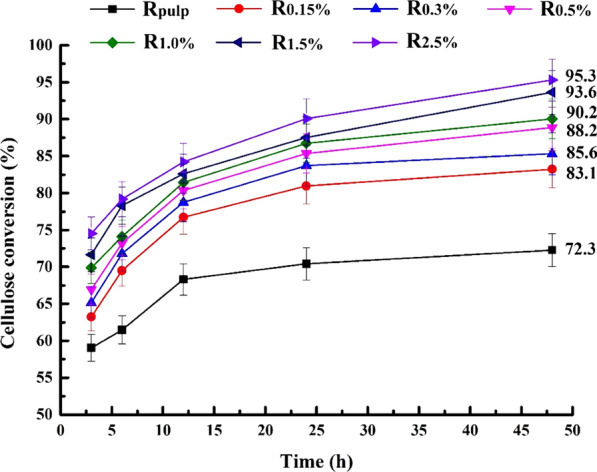
Glucose yields of enzymatic hydrolysis of the delignified ryegrass (R_pulp_) and the cellulose-rich substrates (R_0.15%_, R_0.3%_, R_0.5%_, R_1.0%_, R_1.5%_, and R_2.5%_) obtained by sequential alkaline treatments of the delignified ryegrass

Hemicelluloses are considered as physical barriers for enzyme to attack cellulosic substrate. The effects of fractional removal of hemicellulosic polymers on enzymatic hydrolysis of cellulose-rich substrates are shown in Fig. 3. The delignified ryegrass achieved 59.0% cellulose conversion rate by enzymatic hydrolysis in first 3 h and 72.3% final glucose conversion in 48 h. The enzymatic conversion of cellulose was further enhanced by removal of hemicellulosic polymers. With the decrease content of hemicelluloses in substrates from 32.7 to 19.2%, the glucose yields of enzymatic hydrolysis increased gradually from 59.0 to 74.5% and 72.3 to 95.3%, respectively. The increase of initial enzymatic conversion was ascribed to the fact that sequential alkaline treatments removed hemicelluloses and increased accessibility of material [6]. NaOH pretreatment of Napier grass removes 84% lignin and achieves 94% glucan conversion rate by enzymatic hydrolysis [29]. Pretreatment with ryegrass and surfactant also improves the enzymatic conversion and achieves 87% reducing sugar yield as the maximum [30]. The high glucose yield in this study may be ascribed to the fact that sequential alkaline extraction not only removed hemicelluloses, but also swelled cellulose macromolecule. Swelling of biomass also occurs during alkaline pretreatment of rice husk with 2% NaOH [31]. However, the successively extracted poplar holocellulose has also yielded an increment of cellulose enzymatic conversion and achieved 61.9% cellulose conversion as the maximum [23]. This higher glucose conversion of ryegrass may be ascribed to the structure difference of these two materials.

### Yields, chemical compositions, and molecular weights of hemicellulosic fractions

Hydroxyl ions can swell of cellulose, disrupt intermolecular hydrogen bonds between cellulose and hemicelluloses, and dissolve hemicelluloses. Thus, alkaline extraction is one of the most efficient methods for isolation of hemicellulosic polymers [32]. Besides, alkaline extraction can gradually recover hemicellulosic polymers from lignocellulosic materials depending on components and molecular weights [33]. Yields, chemical compositions, and molecular weights of hemicellulosic fractions are shown in Tables 2 and 3. Sequential alkaline extractions of delignified ryegrass with 0.15, 0.3, 0.5, 1.0, 1.5, and 2.5% KOH solution recovered 7.3, 5.8, 33.9, 13.9, 11.3, and 8.7% hemicelluloses, respectively, equal to 80.9% of total hemicelluloses in holocellulose. It can be seen that the yields of hemicelluloses increased with increasing of alkaline concentration from 0.15 to 0.5%. This result suggested that most hemicelluloses were recovered in the early part of the alkaline extraction procedure. However, a continuous increase of alkali concentration to 2.5% declined yields to 8.7%. This result indicated the degradation of hemicellulosic fractions under alkaline condition, which was consisting with molecular weight of hemicellulosic fractions.

**Table 2:** Yields and sugar compositions (relative %, w/w) of hemicellulosic fractions obtained from sequential alkaline extractions of delignified ryegrass

Samples	Yield^a,c^ (%)	Sugar composition^b,c^ (relative %)						
		Ara	Gal	Glu	Xyl	Man	GluA	GalA
H_0.15%_	7.3 ± 0.3	29.4 ± 0.9	9.4 ± 0.3	10.3 ± 0.3	45.1 ± 1.5	1.1 ± 0.0	2.7 ± 0.0	2.0 ± 0.0
H_0.3%_	5.8 ± 0.2	29.8 ± 1.0	10.2 ± 0.3	8.8 ± 0.2	46.2 ± 1.5	ND	2.0 ± 0.0	3.0 ± 0.1
H_0.5%_	33.9 ± 1.6	28.6 ± 0.9	10.3 ± 0.3	7.8 ± 0.2	48.1 ± 1.5	1.0 ± 0.0	2.5 ± 0.0	1.6 ± 0.0
H_1.0%_	13.9 ± 0.6	29.0 ± 0.9	8.4 ± 0.2	3.7 ± 0.1	52.8 ± 1.6	1.1 ± 0.0	1.8 ± 0.0	3.2 ± 0.1
H_1.5%_	11.3 ± 0.5	22.7 ± 0.7	6.4 ± 0.2	12.8 ± 0.4	54.2 ± 1.7	ND	1.0 ± 0.0	2.9 ± 0.0
H_2.5%_	8.7 ± 0.4	18.3 ± 0.6	3.9 ± 0.1	13.5 ± 0.4	62.5 ± 2.1	ND	0.6 ± 0.0	1.2 ± 0.0

^a^Yields of the hemicelluloses, calculated as [(the weight of the hemicelluloses obtained in each alkaline extraction)/(the weight of the hemicelluloses in the delignified ryegrass)] × 100%;

^b^Ara, arabinose; Gal, galactose; Glu, glucose; Xyl, xylose; Man, mannose; GalA, galacturonic aicd; GluA, glucuronic aicd; ND, not detected

^c^The values are mean ± SD of three parallel determinations

**Table 3:** Weight-average molecular weights (*M*_*w*_) and number-average molecular weights (*M*_*n*_) (g/mol), and polydispersity (*M*_*w*_*/M*_*n*_) of hemicellulosic fractions isolated by sequential alkaline extractions of delignified ryegrass

Samples^a^	*M*_*w*_^b^	*M*_*n*_^b^	*M*_*w*_/*M*_*n*_
H_0.15%_	67,510 ± 3038	39,670 ± 1786	1.70
H_0.3%_	61,480 ± 2829	37,040 ± 1704	1.66
H_0.5%_	55,460 ± 2219	28,730 ± 1150	1.93
H_1.0%_	52,120 ± 2242	25,930 ± 1115	2.01
H_1.5%_	51,420 ± 2366	24,660 ± 1135	2.09
H_2.5%_	50,720 ± 2283	22,480 ± 1012	2.26

^a^Corresponding to the hemicellulosic fractions in Table 2

^b^Molecular weights values (*M*_*w*_ and *M*_*n*_) are mean ± SD of three parallel determinations

The monosaccharide in hemicellulosic polymers is always determined by the neutral sugars and uronic acids released during the acid hydrolysis. Hemicelluloses in ryegrass were fractionated into six fractions by sequential alkaline extractions. It can be seen that xylose was the major neutral sugar of the six hemicellulosic fractions followed by arabinose, glucose, and galactose. Mannose, glucuronic acid, and galacturonic acid were found to be minor amount components in hemicelluloses. As the increase of alkaline concentration, the contents of xylose increased from 45.1 to 62.5%, accompanying with the decrease contents of arabinose and galactose from 29.4 to 18.3%, and from 9.4 to 3.9%, respectively. These phenomena suggested that xylan was the backbone of ryegrass hemicelluloses. Arabinose and minor quantity of uronic acids might substitute on the backbone of xylan as side chains. Besides, the ratio of arabinose to xylose decreased from 0.65 to 0.29, indicating that the linkages between side chains and backbone were cleaved under the alkaline concentration. In addition, glucose was found to be in the third large amount of neutral sugars and its content decreased from 10.3 to 3.7% as alkaline concentration increased from 0.15 to 1.0%. It revealed that *β*-glucan was one of polysaccharides in ryegrass hemicelluloses. However, a further increase of alkaline concentration resulted in an increase of glucose concentration in hemicelluloses. This result might be ascribed the fact that cellulose was degraded during 1.5% and 2.5% KOH extractions. An increment of glucose content in hemicelluloses with increasing of alkaline concentration is also observed in the research of alkaline extraction of *Caragana korshinskii* Kom [34].

Molecular mass is an important parameter which affects physicochemical properties of hemicelluloses. Generally, the molecularly uniformed polysaccharides always have polymerization degrees in excess of 50 and polydispersity below 3 [35]. Table 3 shows the weight-average (*M*_*w*_) and number-average molecular weights (*M*_*n*_) and polydispersity values (*M*_*w*_/*M*_*n*_) of six alkaline hemicelluloses from ryegrass. The *M*_*w*_ of hemicellulosic fractions gradually decreased from 67,510 to 52,120 g/mol as the alkaline concentration rose from 0.15 to 1.0%. It indicated that polysaccharides were degraded under the alkaline condition with the increase of the alkaline concentrations. The polydispersity indexes of hemicelluloses ranged from 1.66 to 2.01, implying a structural homogeneity of all hemicellulosic fractions. Further increase of KOH concentration to 1.5% and 2.5% degraded both hemicelluloses and amorphous cellulose. The co-participation of cellulose fragments and hemicellulosic polysaccharides introduced a slight increase of polydispersity indexes of hemicelluloses from 2.09 to 2.26.

### FT-IR spectra analysis of hemicellulosic fractions

**Fig. 4:**
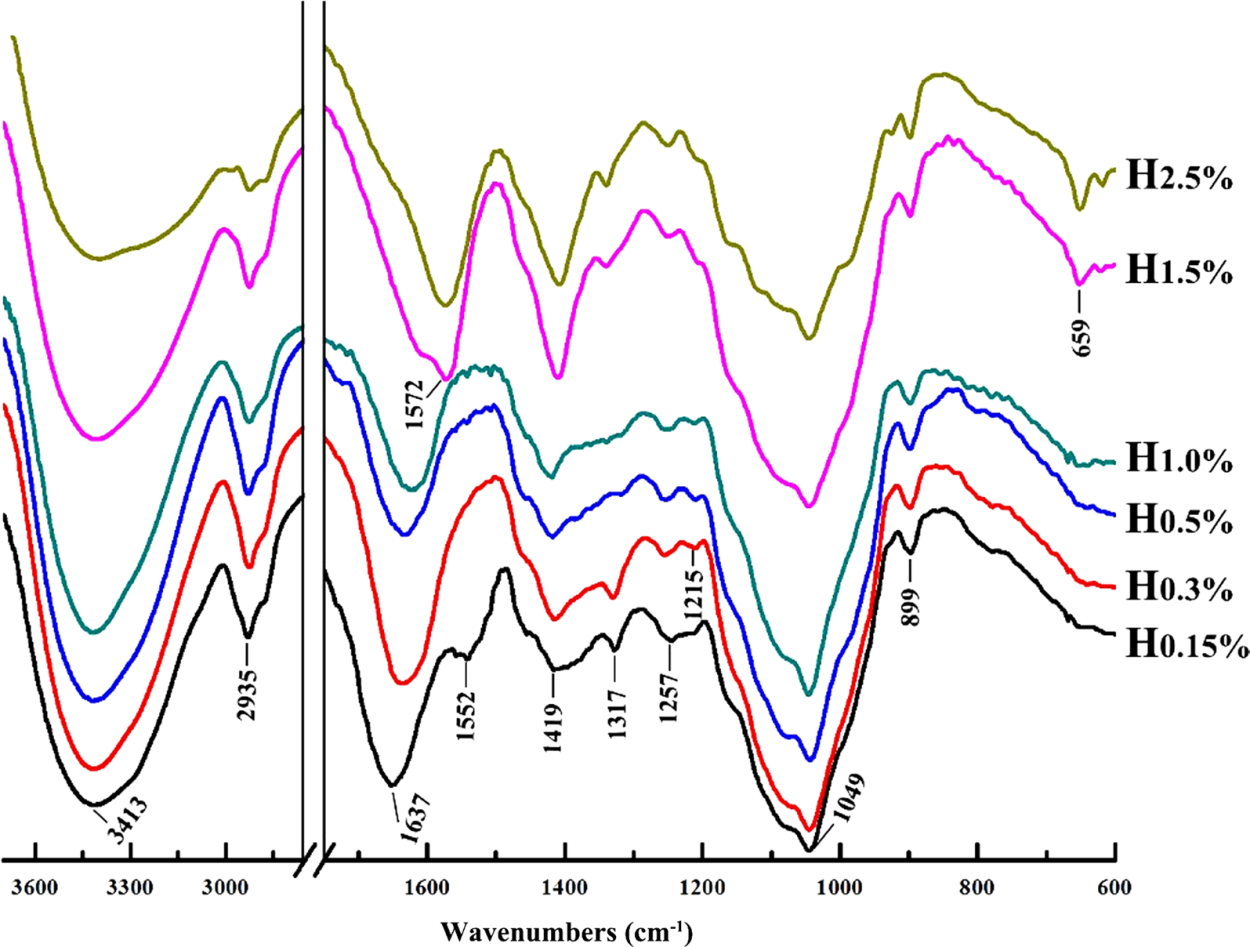
FT-IR spectra of hemicellulosic fractions (H_0.15%_, H_0.3%_, H_1.0%_, H_1.5%_, and H_2.5%_) isolated by sequential alkaline treatments of the delignified ryegrass

FT-IR spectra of hemicellulosic fractions are shown in Fig. 4. The spectra are dominant by signals at 3413 and 2935 cm^−1^ due to stretching vibration of –OH and C–H, respectively. The peaks for O–H in-plane bending occur at 1317, 1257, and 1215 cm^−1^, while O–H out-of-plane bending is observed at 659 cm^−1^. The signals originate from C–O stretching is distributed in the range of 1200–950 cm^−1^, which are fingerprint region of hemicellulosic polysaccharides. The prominent band at 1049 cm^−1^ is attributed to the C–O, C–C stretching, or C–OH bending typical of xylans. The shoulder band at 899 cm^−1^ is attributed to the *β*-linkages of hemicelluloses skeleton. All spectra of hemicelluloses showed similarities in this region, which was consistent with similar sugar components detected in hemicellulosic fractions (Table 2). The massive hydroxyl groups give hemicellulosic polysaccharides strong affinity for water. The band at 1637 cm^−1^ is assigned to the absorption of water on hemicelluloses. The signal at 1419 cm^−1^ is evidence for symmetric stretching of anion carboxylate, originating from salt state of the uronic acids side chain. Besides, the peak at 1552 cm^−1^ in spectrum of H_0.15%_ has a contribution from the associated lignin. However, this absorbance disappeared in spectra of the hemicellulosic fractions obtained from further steps of the alkali extraction with the increasing its concentrations. This result is consistent with the signal for lignin observed in the spectra of cellulose-rich substrates. These phenomena were ascribed the fact that hemicelluloses associated with lignin through chemical bonds and form lignin-carbohydrate complexes (LCC) in plant cell wall [36]. Alkali can effectively cleave the linkages in LCC and promote the dissolution of hemicelluloses. The associated lignin was also determined in the alkali-soluble hemicelluloses from delignified peashrub [37].

### NMR spectra analysis of hemicellulosic fractions

**Fig. 5:**
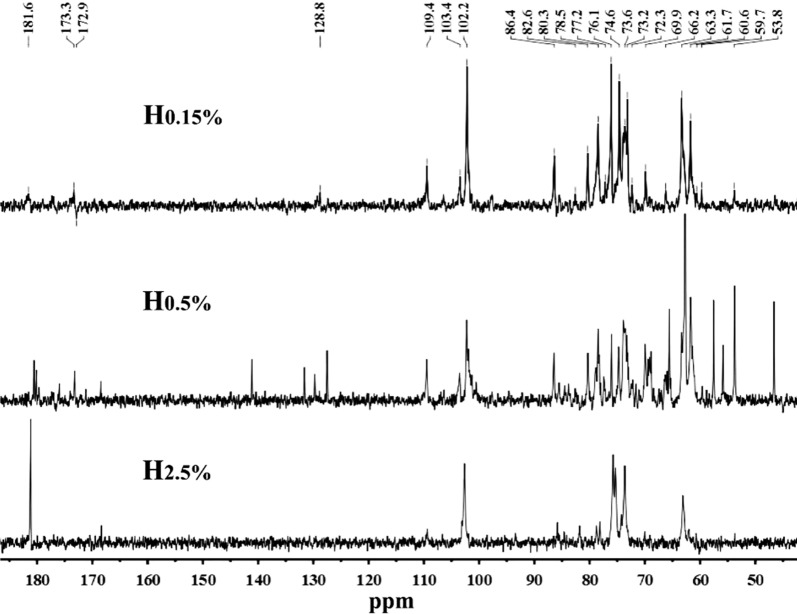
^13^C-NMR spectra of hemicellulosic fractions (H_0.15%_, H_0.5%_, and H_2.5%_) isolated by sequential alkaline treatments of the delignified ryegrass

**Fig. 6:**
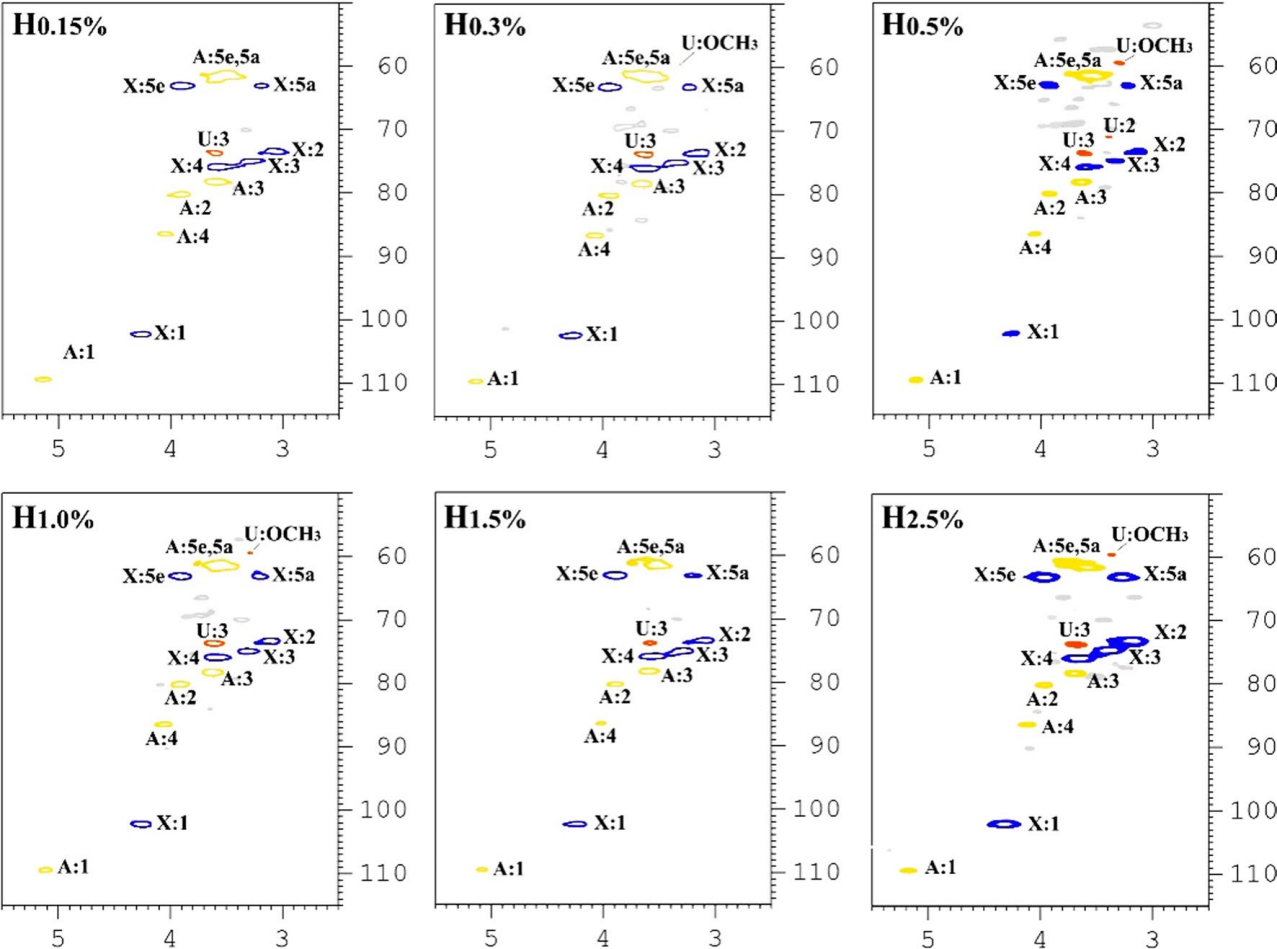
2D-HSQC NMR spectra of hemicellulosic fractions (H_0.15%_, H_0.3%_, H_0.5%_, H_1.0%_, H_1.5%_, and H_2.5%_) isolated by sequential alkaline treatments of the delignified ryegrass

NMR is an efficient technology to assay and identify the backbone and type of sidechain of polymers. The structural characteristics of hemicellulosic fractions were elucidated by ^13^C and HSQC NMR, and are illustrated in Figs. 5 and 6, respectively. The assignment data of HSQC NMR spectra are given in Table 4. The signals for ^13^C-NMR were assigned on the basis of the HSQC spectra and a previous literature [38]. The signals of different structural sugars are overlapped in the ^13^C-NMR spectra. The signals at 102.2, 76.1, 74.6, 73.6, and 63.3 ppm correspond to C_1_, C_4_, C_3_, C_2_, and C_5_ of *β*-(1–4)-linked-d-Xylp units, respectively. The signals for C_1_–C_5_ of arabinose appeared at 109.4, 80.2, 78.5, 86.4, and 61.7 ppm, respectively. The signals observed at 173.3, 82.6, 72.3, and 59.7 ppm are originated from the C_6_, C_4_, C_5_ and methoxyl group of 4-*O*-methyl-d-glucuronic acid, respectively. However, the C_6_ of dissociative glucuronic acid was observed at 181.6 ppm. The present of *β*-glucans in hemicelluloses was identified by the signals at 80.3 ppm (C_3_) and 60.6 ppm. The occurrence of galactose was observed as the signal at 69.0/3.88 ppm in HSQC of spectra. These results implied that the alkaline extract hemicelluloses from ryegrass presumably composed of galactoarabinoxylans, l-arabino-(4-*O*-methyl-d-glucurono)xylans, and *β*-glucans. The results are consisting with structural sugar composition analysis and previous studies [39, 40].

**Table 4:** Assignments of ^13^C-^1^H cross-signals in HSQC spectra of hemicellulosic fractions isolated by sequential alkaline extractions from the delignified ryegrass

Glycosyl	Assignments (ppm)							
		1	2	3	4	5eq^d^	5ax^e^	OCH_3_
X^a^	^13^C	102.4	73.5	75.0	76.0	63.2	63.2	
	^1^H	4.35	3.17	3.32	3.60	3.93	3.21	
U^b^	^13^C		71.2	73.8				59.6
	^1^H		3.40	3.61				3.31
A^c^	^13^C	109.6	80.2	78.3	86.5	61.7	61.7	
	^1^H	5.25	3.91	3.63	4.05	3.73	3.55	
Gal	^13^C		69.5					
	^1^H		3.85					

^a^X, (1 → 4)-*β*-d-Xyl*p*

^b^U, 4-*O*-methyl-*α*-d-Glc*p*A

^c^A, α-ʟ-Araf residues

^d^eq, equatorial

^e^ax, axial

## Conclusions

Cellulose-rich substrates and hemicellulosic fractions were recovered from ryegrass holocellulose by sequential KOH extractions, respectively. With the dissolution of hemicelluloses in alkaline aqueous, the content of hemicelluloses in cellulose-rich substrates decreased from 32.7 to 19.2%, and accompanying decrease of cellulose-rich substrates yields from 100 to 27.7%. Alkaline extraction also removed amorphous cellulose, increasing crystallinity indexes of cellulose. The removal of hemicelluloses also reduced the physical barriers of substrates for enzyme, yielding 1.32-fold enhancement of enzymatic conversion of cellulose-rich substrates. In addition, the hemicellulosic fractions obtained from the sequential alkaline extractions contained arabinoxylans and part of *β*-glucans.

## Materials and methods

### Materials

Ryegrass (35 days old) was harvested from the farm of Guangxi University. It was air dried and ground in a pulverizer. Next, the ryegrass powder was extracted with toluene–ethanol (2:1, v/v) for 5 h to remove wax and chlorophyll, and employed to delignification with NaClO_2_ under acidic condition. The delignified residue was labeled as R_pulp_ and submitted to alkaline extraction for cellulose-rich substrates and hemicellulosic fractions preparation.

### Sequential alkaline extractions and isolation of hemicellulosic fractions

**Fig. 7:**
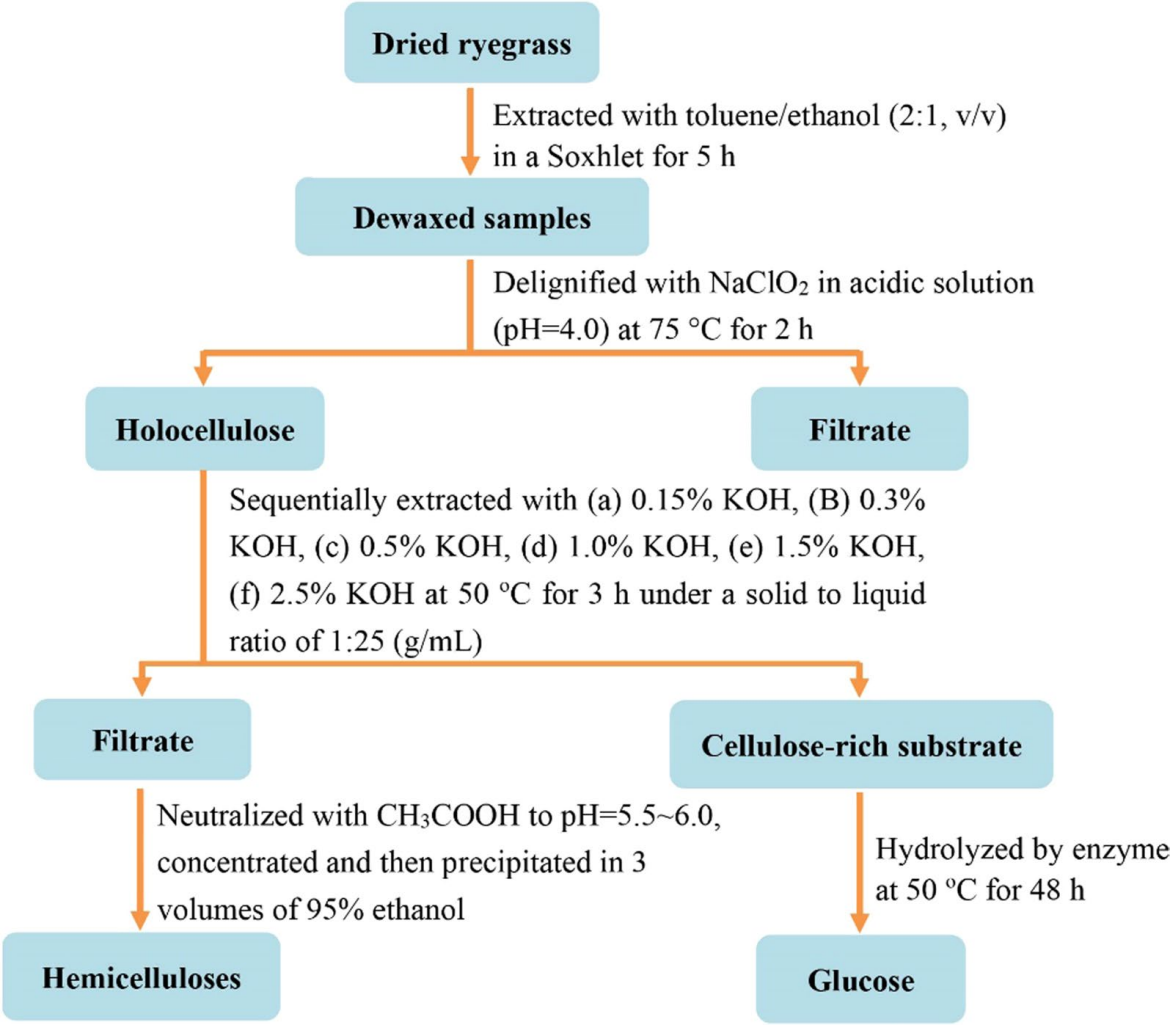
Scheme for preparation of cellulose-rich and hemicellulosic substrates from delignified ryegrass

Sequential alkaline extractions of delignified ryegrass were conducted at a solid–liquid ratio of 1:25 (w/v) with 0.15, 0.3, 0.5, 1.0, 1.5, and 2.5% (w/v) KOH aqueous at 50 °C for 3 h. After incubation, the solid fractions were filtered with a Brinell funnel, washed repeatedly with distilled water, and then oven dried at 55 °C for 16 h. The filtrates were regulated to pH 5.5–6.0 with acetic acid, and vaporized to 30 mL using a vacuum rotary evaporator. The soluble hemicellulosic fractions were obtained by the precipitation of the concentrated aqueous in three volumes of ethanol. Then, the precipitates were recovered by centrifugation and freeze-dried. All the cellulose-rich substrates and hemicellulosic fractions obtain by sequential alkaline extraction were labeled as R_pulp_, R_0.15%_, R_0.3%_, R_0.5%_, R_1.0%_, R_2.5%_, and H_0.15%_, H_0.3%_, H_0.5%_, H_1.0%_, H_1.5%_, and H_2.5%_, respectively, according to the alkali concentration. The separation scheme of cellulose-rich and hemicellulosic fractions is illustrated in Fig. 7. All the extraction experiments were repeated at least in triplicate. The average yields of cellulose-rich and hemicellulosic fractions were given, and the standard deviation (SD) of the three determination was less than 3.3% (Tables 1 and 2).

### Physicochemical characterization of cellulose-rich substrates and hemicellulosic fractions

Chemical components of cellulose-rich substrates and hemicellulosic fractions from the delignified ryegrass were analyzed according to the methods of US National Renewable Energy Laboratory (NREL) [41]. Particularly, the neutral sugars and uronic acids in the samples were analyzed by high-performance anion exchange chromatography (HPAEC), and the molecular weights of hemicellulosic fractions were determined by gel permeation chromatography (GPC) [32]. The analytical experiments were conducted with three parallel performs. The mean values of the chemical composition analysis from the samples are given in Tables 1 and 2, and the SD value of the three parallel performs was less than 2.1%. Meanwhile, the mean values of molecular weights of hemicellulosic fractions are given in Table 3, and the SD value of the three analysis results was less than 3040 (g/mol).

FT-IR spectra of cellulose-rich substrates and hemicellulosic fractions were recorded on a Bruker Tesor 27 FT-IR spectrometer. Each substrate was mixed with spectroscopic grade potassium bromide at a concentration of 1% and ground to a fine powder. Then, the mixtures were subsequently pressed into disks using 10 tons of pressure for 1 min. The spectra of substrates were collected at a resolution of 4 cm^−1^ in the wavelength range of 4000–600 cm^−1^.

^13^C and 2D-HSQC NMR spectra of hemicellulosic polymers were recorded on a Bruker AVIII 400 MHz spectrometer. For NMR spectroscopic experiments, the hemicellulosic samples (80 mg for ^13^C and 20 mg for 2D-HSQC) were dissolved into 0.5 mL D_2_O. The ^13^C-NMR spectra were recorded at 25 °C after 30 000 scans. A 30° pulse flipping angle, a 9.2 μs pulse width, and a 1.36 s acquisition time between scans were used. The spectra widths for HSQC were 5000 and 20 000 Hz for the ^1^H and ^13^C dimensions, respectively. The number of collected complex points was 1024 for the ^1^H dimension with a recycle delay of 5 s. The number of transients for the HSQC spectra was 128, and 256 time increments were always recorded in the ^13^C dimension.

Solid-state cross-polarization/magic angle spinning (CP/MAS) ^13^C-NMR spectra of cellulose-rich substrates were recorded on the spectrometer mentioned above. The dried sample was packed into a 4 mm zirconia (ZrO_2_) rotor, and measurement was performed using a CP pulse program with a 1 ms match time and a 2 s delay between transients. The spinning rate was 5 kHz. Calibration was done externally to the carbonyl carbon of glycine at 176 ppm.

### Enzymatic hydrolysis of cellulose-rich substrates

Enzymatic hydrolysis was executed at 2% substrate (w/v) in 50 mM sodium acetate buffer (pH 4.8) with enzyme loading of 15 FPU/g substrates using a double-layer oscillating incubator at 170 rpm at 50 ºC for 48 h. Commercial cellulase (Cellic® CTec2) was purchased from Novozymes (Beijing, China), which contained 100 FPU cellulase in 1 mL enzyme solution. During enzymatic hydrolysis, 0.2 mL hydrolysates were sampled periodically and analyzed by an HPAEC system. All enzymatic hydrolysis experiments were carried out in triplicate. The average values of the three results are given in Fig. 3 and the SD value was less than 3.5%.

## Data Availability

All data generated or analyzed during this study are included in this published article.
